# Pulmonary Complications in Cirrhosis: Current Concepts and Clinical Perspectives

**DOI:** 10.3390/biomedicines14071499

**Published:** 2026-07-02

**Authors:** Sarocha Vivatvakin, Duangporn Werawatganon

**Affiliations:** Center of Excellence in Alternative and Complementary Medicine for Gastrointestinal and Liver Diseases, Department of Physiology, Faculty of Medicine, Chulalongkorn University, Bangkok 10330, Thailand; sarocha.v@chula.ac.th

**Keywords:** cirrhosis, hepatic hydrothorax, hepatitis C virus, hepatopulmonary syndrome, idiopathic pulmonary fibrosis, liver–lung axis, portal hypertension, portopulmonary hypertension

## Abstract

The liver and lungs maintain an essential anatomical and physiological network crucial for systemic homeostasis. In the presence of cirrhosis, particularly when accompanied by portal hypertension, this intricate communication is disrupted. The resulting alterations can lead to a range of pulmonary complications through intertwined vascular, immunologic, and mechanical mechanisms that underscore the close relationship between these two organs. This review provides an overview of the liver–lung axis and summarizes current concepts of the pathophysiological processes by which advanced liver disease contributes to major respiratory complications. It also highlights diagnostic principles and clinical manifestations, with emphasis on hepatopulmonary syndrome, portopulmonary hypertension, and hepatic hydrothorax. Although these conditions differ substantially in their underlying pathogenesis, they share common clinical consequences, including impaired arterial oxygenation, reduced functional capacity, and an increased risk of mortality prior to liver transplantation. In addition, the review explores the ongoing debate regarding the potential association between chronic hepatitis C virus infection and pulmonary fibrosis. Overall, early recognition of these pulmonary complications is crucial, as they have important implications for symptom burden, therapeutic decision-making, liver transplant eligibility, and overall clinical outcomes in patients with chronic liver disease.

## 1. Introduction

The liver and lungs function in a coordinated manner to support essential physiological activities that preserve internal balance. Their circulatory systems are closely linked, with hepatic and pulmonary blood flow forming an interconnected network that contributes to systemic homeostasis [1]. Cirrhosis is histopathological defined by loss of the normal hepatic architecture, with diffuse regenerative nodules encased by dense fibrous septa. This process causes parenchymal extinction and marked distortion of both the liver structure and intrahepatic vascular architecture. As a result, resistance to portal venous blood flow increases, contributing to portal hypertension and progressive hepatic failure. Major causes of cirrhosis include alcohol-related liver disease, metabolic and genetic conditions such as metabolic dysfunction-associated steatotic liver disease (MASLD), autoimmune liver diseases, drug- and toxin-induced injury, and chronic infections, particularly hepatitis B and hepatitis C [2]. As effective communication between the liver and lungs is essential for host defense against pulmonary bacterial infections, in patients with cirrhosis and portal hypertension, the liver–lung communication becomes disrupted as portosystemic shunting allows substances including gut-derived metabolites, vasoactive mediators, and toxins that would normally undergo hepatic clearance to enter the systemic and pulmonary circulations resulting in vascular remodeling and diseases [3]. These changes along with other consequences of cirrhosis may contribute to several clinically important pulmonary complications, including hepatopulmonary syndrome (HPS), portopulmonary hypertension (PoPH), and hepatic hydrothorax (HH). These liver-related pulmonary complications should be differentiated from primary respiratory disorders, which may occur concomitantly in patients with liver disease but do not arise as a consequence of cirrhosis or portal hypertension. In addition, the relationship between chronic hepatitis C virus infection, one of the cirrhosis etiologies, and pulmonary fibrosis remains controversial, with epidemiologic associations but limited evidence for direct causality. Despite advancing clinical awareness, important gaps remain in our understanding of precise pathophysiological mechanisms driving the development of these complications. This review was therefore motivated by the need to integrate current pathophysiologic concepts with practical clinical perspectives, emphasizing development of novel treatment and improvement of patient outcomes.

## 2. Liver–Lung Axis

The liver, unlike other organs, receives dual vascular blood supply consisting of 25% from the hepatic artery and 75% from the portal vein. The portal vein receives drainage mainly from venous outflow of the gastrointestinal tract, formed by the union of the superior mesenteric vein and the splenic vein [4]. Gut-derived metabolites are absorbed by enterocytes and enter the portal circulation. After the first-pass metabolism by the liver, they enter the systemic circulation and are distributed to all organs including the lungs. With the large surface area of the blood–gas interface of the lungs, they are highly effective in receiving circulating signals [5]. Another pathway of transferring immunomodulatory molecules, lipids, and immune cells is the mesenteric–thoracic duct pathway. The intestine is drained by the mesenteric system, unlike the portal vein, and mesenteric lymph nodes drain into the cisterna chyli, subsequently into the thoracic duct, and finally mix into the left subclavian vein bypassing the liver entirely [6].

The liver plays a central immunoregulatory role by serving as a major reservoir of immune cells including macrophages, natural killer cells, and T lymphocytes, and by producing key mediators such as complement proteins and C-reactive protein. In addition, it secretes a wide range of hormones, cytokines, chemokines, and liver-derived signaling molecules known as hepatokines, which coordinate systemic immune responses. These hepatokines such as PCSK9 and hepcidin, along with factors released by hepatic leukocytes into the circulation, interact with the pulmonary vasculature to regulate immune cell activation, proliferation, differentiation, and trafficking within the lungs. Through these mechanisms, liver-derived signals help maintain pulmonary vascular integrity and right heart function, while directing immune processes [7].

During normal circumstances, portal circulation functions under a low-pressure state. The portal vein supplies the liver, where first-pass metabolism occurs before entering the systemic circulation via the hepatic vein [4]. Also, there are the portosystemic anastomoses which connect with various sites such as the esophagus, rectum, umbilical, and colonic vein in which a small amount of blood bypasses the liver, the so-called “portosystemic shunt” [8]. Due to the low-pressure state of the portal vein, all potential shunts from these anastomoses are closed [9,10].

When portal hypertension develops, whether due to cirrhotic or non-cirrhotic causes, if the pressure in the portal system exceeds the systemic circulation, portosystemic shunting increases. This results in a greater proportion of blood bypassing the liver and entering the systemic circulation without undergoing hepatic metabolism [8]. Consequently, substances such as gut-derived antigens, toxins, microbial products, vasoactive mediators, inflammatory cytokines, and other hazardous substances can pass directly into the systemic circulation without detoxification [11]. These unmetabolized compounds will be transferred to the pulmonary circulation which can trigger or exacerbate parenchymal and vascular remodeling, ultimately contributing to the development of pulmonary diseases [8]. Furthermore, portal hypertension leads to splanchnic and intestinal venous congestion, which results in alteration of the gut microbiome and acceleration of bacterial and metabolite translocation [12].

Mostly well-known complications from the pulmonary point-of-view regarding chronic liver disease or cirrhosis include HPS, PoPH, and HH. The information regarding these conditions is summarized in Table 1.

## 3. Hepatopulmonary Syndrome (HPS)

Alterations in pulmonary function associated with liver disease have been recognized for over a century; however, the term “hepatopulmonary syndrome” was not introduced until 1977, following the identification of intrapulmonary vasodilatations (IPVDs) as a key factor contributing to impaired gas exchange [13]. This condition can also be found in both cirrhotic and non-cirrhotic portal hypertension including portal vein thrombosis, Budd–Chiari syndrome, and diseases with vascular abnormalities characterized by altered blood flow between the liver and lung such as cavopulmonary shunts and Abernethy malformation [14,15]. The severity of the syndrome is not directly correlated with the severity or cause of the underlying liver disease [16]. These IPVDs whether pre-capillary or capillary vessels are common in patients with advanced chronic liver disease, with an estimated prevalence of 13–56% [17].

### 3.1. Pathogenesis and Pathophysiology

**Figure 1:**
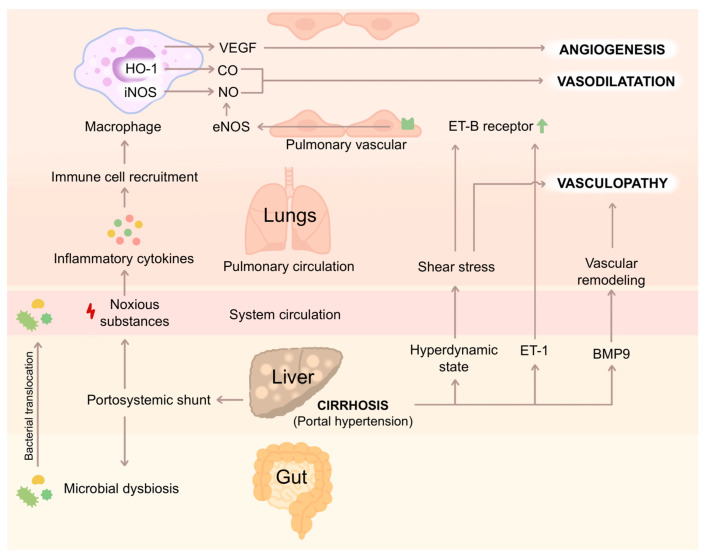
Hepatopulmonary syndrome pathogenesis. Vasodilatation: Microbial dysbiosis and bacterial translocation along with noxious substances are introduced into the systemic circulation via portosystemic shunts. These trigger the release of inflammatory cytokines and subsequent immune cell recruitment. Activated macrophages upregulate iNOS and HO-1, releasing potent vasodilators like NO and CO, respectively. Simultaneously, a hyperdynamic circulatory state and increased pulmonary shear stress lead to upregulation of pulmonary ET-B receptors, which enhances eNOS activity, further exacerbating vasodilatation. Angiogenesis: Recruited macrophages secrete pro-angiogenic mediators, most notably VEGFs. These factors stimulate abnormal vascular proliferation and the formation of intrapulmonary shunts. Vasculopathy: Disrupted hepatic synthesis of BMP9 impairs pulmonary vascular homeostasis, which drives aberrant vascular remodeling and contributes to overall vasculopathy. Green arrow mean increased. BMP9, bone morphogenetic protein 9; CO, carbon monoxide; eNOS, endothelial nitric oxide synthase; ET-B, endothelin-B; ET-1, endothelin-1; HO-1, heme oxygenase-1; iNOS, inducible nitric oxide synthase; NO, nitric oxide; VEGF, vascular endothelial growth factor.

The primary pathophysiological feature of HPS is dilation of both pre-capillary and capillary intrapulmonary vessels. The pathogenesis of HPS is illustrated in Figure 1.

The first mechanism is driven by various vasodilators [18]. In chronic liver disease, bacterial translocation occurs due to intestinal bacterial overgrowth, disruption of the mucosal barrier, reduced hepatic clearance, and the presence of portosystemic shunts. As a result, bacterial products and endotoxins can enter the pulmonary circulation, where they trigger the release of inflammatory mediators such as tumor necrosis factor-alpha (TNF-α), interleukin-1 (IL-1), interleukin-6 (IL-6), and chemokines. This promotes the recruitment of immune cells to the lungs through increased expression of chemotactic receptors, including CX3CR1 and CXCR2 [19]. Subsequent monocyte activation further enhances nitric oxide-mediated vasodilation via inducible nitric oxide synthase (iNOS) and stimulates the production of other vasodilators, such as carbon monoxide via heme oxygenase-1 (HO-1) [15,19]. Endothelin-1 (ET-1) levels are elevated in liver cirrhosis due to increased hepatic production. When released from vascular endothelial cells and acting on endothelin A (ET-A) receptors on vascular smooth muscle, or when administered systemically at pharmacologic doses, ET-1 produces potent vasoconstriction. However, ET-1, in the condition of HPS, activates endothelin B (ET-B) receptors on endothelial cells and enhances endothelial nitric oxide synthase (eNOS) activity, increasing nitric oxide (NO) production. NO then diffuses into adjacent smooth muscle cells, stimulating the guanylate cyclase–cGMP pathway and resulting in vasodilation. Furthermore, in the hyperdynamic circulatory state and increased pulmonary shear stress, upregulation of endothelial ET-B receptors occurs leading to worsening of HPS [20].

The second proposed mechanism involves angiogenesis and the formation of intrapulmonary shunts [21]. In HPS, increased pulmonary expression of pro-angiogenic factors such as von Willebrand factor (vWF), endoglin, vascular endothelial growth factor (VEGF), platelet-derived growth factor (PDGF), and placental growth factor (PlGF) has been observed [19,21]. These mediators, particularly those produced by infiltrating monocytes, are thought to contribute to abnormal vascular proliferation and the development of intrapulmonary shunting [21].

The third mechanism involves a protein called bone morphogenetic proteins (BMPs) which are key signaling molecules involved in the pathobiology of pulmonary vascular stability, including BMP9, as this protein is synthesized in the liver [22,23]. In patients with underlying liver disease, disruption of BMP9 production or function may impair vascular homeostasis, thereby contributing to the development of pulmonary vascular remodeling and vasculopathy [22,23].

**Figure 2:**
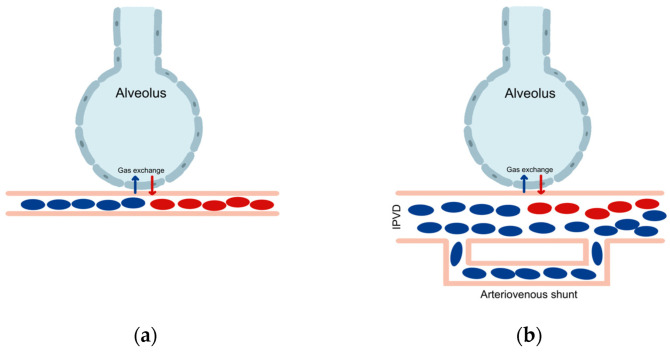
Pathophysiology of gas exchange abnormalities in hepatopulmonary syndrome. (**a**) Normal oxygen exchange at alveolar and pulmonary capillary interface; (**b**) impaired gas exchange in hepatopulmonary syndrome caused by IPVDs including V/Q mismatch due to increased pulmonary perfusion with normal ventilation, diffusion defect due to increased distance from vasodilatation and increased capillary transit time, and arteriovenous shunt resulting in mixing of venous and arterial blood leading to hypoxemia. Blue color mean poorly oxygenated red blood cells; Red color mean fully oxygenated red blood cells. IPVD, intrapulmonary vasodilatation; V/Q, ventilation–perfusion.

The HPS’s pathophysiology can be understood through three interrelated mechanisms which is demonstrated in Figure 2 [24,25].

First, the ventilation and perfusion (V/Q) mismatch occurs when there is increased pulmonary blood flow due to vasodilatation with unchanged ventilation [24]. If severe, a right-to-left shunt will occur [24].

Second, a diffusion defect will occur as oxygen is unable to complete the diffusion process during the normal blood–gas interface time [25]. As the dilation of pulmonary capillaries increases, the distance between alveolar gas and red blood cells also increases [26]. Furthermore, combined with increased pulmonary blood flow from the hyperdynamic state and reduced capillary transit time, this prevents full equilibration of oxygen before blood leaves the pulmonary circulation, contributing to an elevated alveolar–arterial gradient [27].

Third, severe intrapulmonary vascular dilatation may lead to portopulmonary venous malformation and true arteriovenous communications resulting in poorly oxygenated blood entering the systemic circulation and causing hypoxemia that responds poorly to supplemental oxygen [24].

### 3.2. Clinical Manifestations

HPS typically presents with the insidious onset of dyspnea, particularly on exertion or even asymptomatically [28]. A pathognomonic feature is the combination of platypnea defined by worsening of breathlessness when shifting from a supine to an upright position and orthodeoxia defined by a drop of ≥5% or ≥4 mmHg in partial pressure of arterial oxygen (PaO_2_) when standing, though these specific signs are present in only about 18–20% of cases [19,29]. Other common physical signs include digital clubbing, cyanosis, fatigue, and spider nevi [28].

### 3.3. Diagnosis

The diagnosis relies on the European Respiratory Society Task Force demonstrating a classic triad including the presence of liver disease or portal hypertension; an impaired gas exchange defined by low arterial oxygenation (PaO_2_ ≤ 80 mmHg) with an elevated alveolar–arterial oxygen gradient ≥ 15 mmHg or ≥20 mmHg for those over 64 years of age in an upright position; and evidence of intrapulmonary vascular dilatations [30,31]. Clinicians usually screen with pulse oximetry, followed by arterial blood gas analysis to confirm hypoxemia.

IPVDs are mainly identified using contrast-enhanced transthoracic echocardiography [32]. Agitated saline is injected intravenously to generate microbubbles, >10 μm in diameter, that normally remain confined to the right heart, as they cannot traverse the pulmonary capillary bed which has the diameter of 8–15 μm but may increase to 500 μm in HPS [32,33]. The appearance of these microbubbles in the left heart after a delay of three or more cardiac cycles suggests the presence of IPVDs or intrapulmonary shunting. If the microbubbles appear in the left heart within three cycles, it is an implication for an intracardiac shunt [34].

A technetium-99m macroaggregated albumin (99mTc-MAA) lung perfusion scan, even less standardized due to its low sensitivity, can also be used to quantify the degree of intrapulmonary shunting [35]. Normally, the injected particles are trapped in pulmonary capillaries, but in the presence of shunts, they bypass the lungs and accumulate in organs such as the brain and kidneys. The uptake of more than 6% by the brain is defined as positive for a shunt [32]. While this method can quantify shunting, it cannot distinguish between intracardiac and intrapulmonary causes [34].

### 3.4. Treatments

Currently, there is no effective medical treatment for HPS other than supplemental oxygen to alleviate hypoxemia. The transjugular intrahepatic portosystemic shunt (TIPS) has been proposed as a strategy to reduce portal pressure in patients with HPS. However, available evidence remains insufficient. In addition, TIPS may worsen the hyperdynamic circulation and potentially enhance pulmonary vasodilation. Therefore, its benefit in adult HPS remains uncertain, and cannot currently be recommended as a treatment for HPS.

Liver transplantation is the only proven curative therapy, completely resolving the gas exchange abnormalities in the vast majority of patients over time including those with severe hypoxemia [36]. Patients with moderate severity of HPS, defined as PaO_2_ ≥ 60 to <80 mmHg, clinically followed-up may be considered, whereas those with HPS-related severe hypoxemia with PaO_2_ < 60 mmHg should receive expedited consideration for liver transplantation and may qualify for MELD exception points before the progression of hypoxemia occurs [14,31]. Because very severe HPS, particularly PaO_2_ < 50 mmHg, is associated with increased post-transplant mortality, arterial blood gas analysis should be repeated every 6 months to reassess severity and facilitate transplant prioritization [37]. Current guideline recommendations support MELD exception allocation for severe HPS, with consideration of additional prioritization in patients with very severe hypoxemia according to United Network for Organ Sharing (UNOS) post-liver transplantation data analysis [37].

## 4. Portopulmonary Hypertension (PoPH)

Pulmonary arterial hypertension (PAH) occurring in the setting of portal hypertension was initially reported in 1951 by Mantz and the term portopulmonary hypertension (PoPH) was later introduced [38]. PoPH is classified as group 1 PAH occurring in the setting of unexplained pre-capillary pulmonary hypertensions with portal hypertension or portosystemic shunt, with or without underlying cirrhosis [39]. It is defined hemodynamically by a mean pulmonary artery pressure (mPAP) > 20 mmHg, a pulmonary artery wedge pressure < 15 mmHg, and elevated pulmonary vascular resistance (PVR) > 2 [39]. PoPH has a prevalence of 2% to 6% among patients with portal hypertension [40], while PoPH accounts for approximately 5% to 15% of cases of group 1 pulmonary hypertension [39,41]. Without liver transplantation or targeted PAH therapy, PoPH carries a catastrophic prognosis, ultimately leading to right ventricular failure [42].

### 4.1. Pathogenesis and Pathophysiology

**Figure 3:**
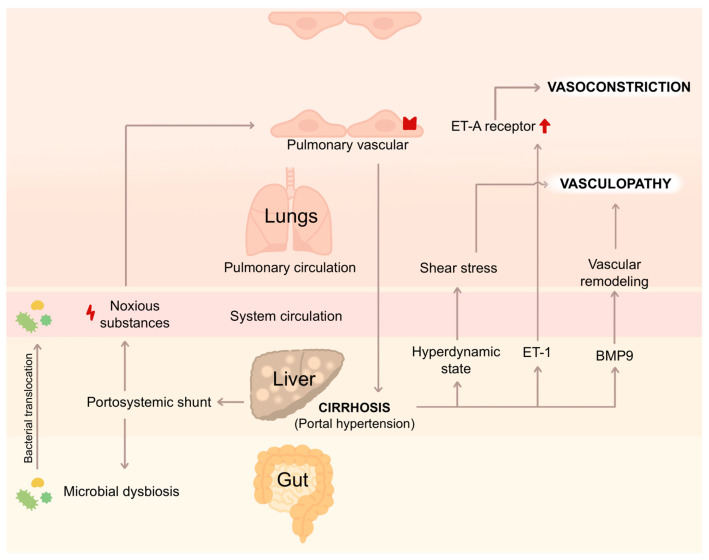
Portopulmonary hypertension pathogenesis. Vasculopathy: Hyperdynamic circulatory state consequences of portal hypertension characterized by splanchnic vasodilation and high cardiac output lead to increase in pulmonary blood flow which generates turbulent flow and elevated shear stress. This shear stress, combined with the effects of BMP9 signaling, impairs vascular integrity and drives smooth muscle proliferation, resulting in abnormal vascular remodeling. Vasoconstriction: Bacterial translocation and noxious substances directly bypass hepatic clearance by portosystemic shunting along with vasoactive mediators like ET-1, resulting in pulmonary vasoconstriction. The interaction between the hepatic and pulmonary systems is reciprocal. Elevated pulmonary arterial pressures can transmit backward, contributing to right-sided heart strain, hepatic congestion, and the further deterioration of liver function. Red arrow mean increased. BMP9, bone morphogenetic protein 9; ET-A, endothelin-A; ET-1, endothelin-1.

The pathophysiological mechanisms underlying the development of PoPH remain incompletely understood. PoPH can arise in both cirrhotic and non-cirrhotic portal hypertension, including conditions such as Budd–Chiari syndrome, portal vein thrombosis, and schistosomiasis. In addition, several other disorders may contribute to pulmonary hypertension in this population, such as cirrhotic cardiomyopathy, pulmonary embolism, and intrahepatic arteriovenous fistulas [43]. The severity of liver disease does not predict either the occurrence or the degree of PoPH. Likewise, previous research has not demonstrated a meaningful relationship between the hepatic venous pressure gradient and pulmonary vascular resistance (PVR) [44]. Other possible underlying mechanisms of PoPH are shown in Figure 3.

One proposed mechanism involves a hyperdynamic circulatory state resulting from increased portosystemic shunting [45]. Splanchnic vasodilation leads to a high cardiac output and salt water retention, which dramatically increases pulmonary blood flow [45]. This leads to turbulent blood flow and elevated shear stress within the pulmonary vasculature, which can impair vascular integrity, promote endothelial dysfunction, increase smooth muscle cell proliferation and drive vascular remodeling and concentric intimal fibrosis, along with reduced nitric oxide bioavailability [1,45]. A significant relationship has also been observed between the presence of large portosystemic shunts and the severity of PoPH, indicating that a hyperdynamic circulatory state may play an important role in its pathogenesis [46].

Another possible cause is relying on BMP9 as well as HPS but with a weaker association [22,23].

Furthermore, noxious substances including toxins and inflammatory mediators that bypass the liver from this shunt also lead to injury of the pulmonary vasculature and pulmonary vasoconstriction. Vasoactive substances including ET-1 bypass the liver and enter the lungs resulting in pulmonary vascular vasoconstriction and an increase in PVR [47].

Also, the portal venous system carries high concentrations of microbial metabolites derived from the gut microbiota, which comprises approximately 10^11^–10^12^ microorganisms [48,49,50,51]. These microbial products can modulate immune cell function not only locally but also in distant organs, including the lungs. Consequently, alterations in the gut microbiome have been linked to PAH. However, no studies to date have specifically investigated the direct role of the gut microbiome in PoPH [4]. Moreover, the interaction between the lungs and liver is bidirectional. As PoPH develops, elevated pulmonary pressures can transmit backward, contributing to hepatic congestion and further deterioration of liver function [52].

### 4.2. Clinical Manifestations

PoPH is frequently asymptomatic in its early stages, but as the disease progresses, patients most commonly complain of progressive dyspnea on exertion and exercise tolerance. More advanced disease manifests with signs of right-sided heart failure, including peripheral edema, ascites, chest pain, syncope, jugular venous distention, and an accentuated, split-second heart sound [53].

### 4.3. Diagnosis

Screening for PoPH in patients with portal hypertension is recommended in patients with signs or symptoms suggestive of pulmonary hypertension. However, routine universal screening for PoPH in asymptomatic patients remains controversial and is not currently recommended, except in those being evaluated for liver transplantation or transjugular intrahepatic portosystemic shunt (TIPS) placement [54]. This is particularly important because moderate-to-severe PoPH may contraindicate liver transplantation, as discussed in more detail below [39].

Doppler echocardiography is the standard screening tool for pulmonary hypertension, as it estimates right ventricular systolic pressure (RVSP) and identifies other echocardiographic signs suggestive of pulmonary hypertension. However, in patients with cirrhosis, a hyperdynamic circulation and elevated cardiac output may increase tricuspid regurgitation velocity (TRV), leading to overestimation of pulmonary artery pressure (PAP) [39]. Therefore, echocardiographic findings alone may result in false positive diagnosis of PoPH. In the study by Krowka et al., among liver transplant candidates with RVSP > 50 mmHg, approximately one-third had normal PVR on right heart catheterization [55]. Thus, patients with RVSP > 50 mmHg or other echocardiographic features suggestive of pulmonary hypertension should undergo right heart catheterization to confirm PoPH and distinguish true pulmonary arterial hypertension from high-flow states, volume overload, or left-sided cardiac dysfunction [39].

### 4.4. Treatments

In patients with a confirmed diagnosis of PoPH, treatment should generally follow the same principles used for other forms of pulmonary arterial hypertension, particularly those with mPAP more than or equal to 35 mmHg [14,39]. Treatment for PoPH relies heavily on PAH-targeted medical therapies. In principle, all therapies approved for PAH may be considered in the treatment of PoPH, although these patients are typically excluded from major clinical trials [39]. Despite this limitation, evidence from multiple studies supports the use of standard PAH-targeted therapies in individuals with PoPH [39,40]. In a 2020 cohort of 637 PoPH patients, most had mild liver disease, with Child–Pugh class A cirrhosis and a median MELD score of 11. The majority were treated with monotherapy, most commonly a phosphodiesterase-5 inhibitor (PDE5i) (sildenafil and tadalafil) or an endothelin receptor antagonist (ERA) (bosentan, ambrisentan, and macitentan), while a smaller proportion received combination therapy. After a median treatment duration of 4.5 months, significant improvements were observed in functional class (FC), 6 min walk distance (6MWD), and PVR. Overall, 5-year survival was 51%, but survival was substantially higher among patients who underwent liver transplantation, reaching 81% at 5 years [40]. A recent systematic review and meta-analysis of 26 studies involving 1019 patients further supported the benefit of PAH-targeted therapy including PDE5i, ERA, and prostanoids in PoPH, demonstrating improvements in survival and pulmonary hemodynamics, including mPAP and PVR. Importantly, medical therapy improved hemodynamics sufficiently to allow approximately 44% of treated patients to become eligible for liver transplantation, and about half were able to discontinue PAH therapy after transplantation [56]. Furthermore, the only randomized controlled trial of PAH-specific therapy in PoPH is the PORTICO trial in which macitentan showed significant reduction in PVR by 35% compared with placebo over 12 weeks in patients with WHO functional class II–III. The medication revealed no major hepatic safety concerns, although peripheral edema was reported [57]. Similarly, an open-label multicenter trial of ambrisentan in treatment-naive patients with Child–Pugh class A or B cirrhosis for 24 weeks also showed significant reduction in PVR and improvement in hemodynamic parameters and WHO FC, although 6MWD did not change significantly. Peripheral edema led to treatment discontinuation in some patients [58]. In summary, PAH-targeted therapies are effective in improving hemodynamics, thereby facilitating safer liver transplantation and contributing to favorable post-transplant survival outcomes.

PoPH itself is not an indication for liver transplantation. Rather, liver transplantation should be considered based on the severity of the underlying liver disease. Meanwhile, moderate-to-severe pulmonary hypertension may represent a contraindication if pulmonary hemodynamics remain inadequately controlled. The International Liver Transplant Society has proposed specific hemodynamic targets for patients with PoPH including a mPAP < 35 mmHg with PVR < 5 Wood units, or alternatively, an mPAP ≥ 35 mmHg with PVR < 3 Wood units with a higher risk of morbidity and mortality. In contrast, an mPAP ≥ 45 mmHg is considered an absolute contraindication to liver transplantation [14].

## 5. Hepatic Hydrothorax (HH)

Pleural effusion related to liver disease was first described in the 19th century by René Laennec, and the term “hepatic hydrothorax (HH)” was later introduced in 1958 [59]. HH is defined as the accumulation of a transudative pleural effusion by Light criteria in patients with liver cirrhosis and portal hypertension, occurring in the absolute absence of primary cardiopulmonary, pleural, or malignant disease. It affects 5% to 16% of patients with decompensated cirrhosis [60].

### 5.1. Pathogenesis and Pathophysiology

HH results from the one-way movement of ascitic fluid into the pleural space through micro- or macroscopic diaphragmatic defects, a process driven by negative intrathoracic pressure and positive intra-abdominal pressure due to ascites [61]. It develops when the volume of fluid entering the pleural cavity exceeds the capacity of the pleural lymphatic system to remove it. Because the right hemidiaphragm is structurally thinner and less muscular than the left, these defects predominantly occur on the right, leading to right-sided pleural effusions in 70% to 85% of cases, while 13–17% and 2–10% of cases present with a left-sided or bilateral effusion, respectively [53,62]. HH can manifest without clinically detectable ascites; this happens when the rate of ascites formation perfectly equilibrates with the rate at which fluid is sucked into the pleural cavity, effectively hiding the abdominal source of the fluid [62]. Furthermore, hypoalbuminemia secondary from chornic liver disease worsens the process of hepatic hydrothorax. If the pleural effusion is massive, compression of lung tissue might occur [63].

### 5.2. Clinical Manifestations

HH can be asymptomatic if the effusion is small, but larger fluid accumulations lead to shortness of breath, non-productive cough, pleuritic chest pain, and hypoxemia. If the static pleural fluid becomes infected which is a condition known as spontaneous bacterial empyema (SBEM), patients may rapidly develop fever, abdominal pain, or encephalopathy [64].

### 5.3. Diagnosis

Diagnosis requires identifying a transudative pleural effusion in a cirrhotic patient, after strictly ruling out primary cardiac or lung diseases as mentioned above. Imaging like chest X-rays are able to identify the effusion, while a diagnostic thoracentesis confirms the transudative nature of the fluid, showing a serum-to-pleural fluid albumin gradient > 1.1 g/dL. As water absorption in the pleural cavity may respond to a slightly higher total protein and albumin compared with ascites fluid, a pleural fluid polymorphonuclear cell count > 250 cells/mm^3^ with a positive culture or >500 cells/mm^3^ with a negative culture confirms the diagnosis of SBEM [60]. Nuclear scans are highly specific for diagnosing hepatic hydrothorax. By injecting a radioactive tracer including 99mTc-human serum albumin or 99mTc-sulphur colloid into the abdomen, this can confirm the diagnosis if the tracer migrates into the chest cavity even in patients without ascites. Additionally, modern imaging tools like computed tomography (CT), magnetic resonance imaging (MRI), and ultrasound are now used to get a detailed look at the actual tears in the diaphragm causing the issue [64].

### 5.4. Treatments

The primary treatment consists of a strict low-sodium diet and diuretics such as spironolactone and furosemide to reduce overall fluid volume [65]. However, pleural effusion may persist despite medical therapy and fluid restriction, eventually progressing to refractory HH [65]. For rapid symptomatic relief in refractory and symptomatic cases, therapeutic thoracentesis is effective, though frequent tapping increases the risk of complications like pneumothorax, bleeding, or infection [66]. Ultimately, much like HPS, liver transplantation is the only definitive, curative treatment for refractory HH [42]. TIPS has been used effectively as either a definitive treatment or a bridge to liver transplantation in patients with refractory HH [37]. A meta-analysis of six studies involving 198 patients with refractory HH showed that TIPS achieved a complete response in 55.8% of patients. These findings suggest that TIPS provides a clinically meaningful response in refractory HH and should be considered early [67]. However, TIPS is only indicated for a specific sub-population with no severe hepatic encephalopathy or right-sided heart failure [66]. Pleurodesis, an alternative treatment, performed using various substances including tetracyclin, talc, or bleomycin through intercostal drainage or pleuroscopy is reserved for patients with refractory HH in the absence of ascites who are not candidates for TIPS or liver transplantation [37,65]. However, the frequent occurrence of procedure-related complications limits its usage.

## 6. Hepatitis C Virus (HCV) and Pulmonary Fibrosis

Hepatitis C virus (HCV), a ribonucleic acid (RNA), is the primary hepatotropic virus responsible for chronic liver diseases, but its lymphotropic nature also leads to numerous systemic extrahepatic manifestations affecting organs such as the heart and lungs [68]. Among these pulmonary complications, idiopathic pulmonary fibrosis (IPF), a chronic, progressive, and often fatal interstitial lung disease, has been heavily investigated as a potential consequence of chronic HCV infection [68].

The epidemiological link between HCV and IPF has been highly debated, with conflicting findings based on geographical and methodological differences. Ueda et al. found a high prevalence of anti-HCV antibodies (28.8%) in Japanese patients with IPF compared to age-matched controls (3.66%) [69]. Similarly, research in Italian cohorts demonstrated increased rates of anti-HCV-positive antibodies (13.3%) in IPF patients compared with a blood donor control group (0.3%) [70]. However, when compared with non-interstitial lung disease including chronic obstructive lung disease, neuromuscular chest wall diseases, pulmonary vascular diseases, bronchiectasis, chronic pleural diseases, and tuberculosis, there is no significant difference [70]. Furthermore a large retrospective cohort study in HCV-positive patients revealed cumulative rates of IPF development were 0.3% at the 10th year and 0.9% at the 20th year particularly in patients over 55 years of age, those with a heavy smoking history, and those with underlying liver cirrhosis, while none of the patients developed IPF in an HBV group [71]. However, from Irving’s study in England, no significant increase in anti-HCV antibody positivity in patients with IPF was found [72]. Another study from Egypt in 2015 also showed that in IPF patients, there was no significant difference in hepatitis C surface antigen (HCVsAg) detection (30%) compared with control (28.3%). However, the study still showed that the presence of HCV is associated with increased severity of the IPF condition [73].

### 6.1. Pathogenesis and Pathophysiology

#### 6.1.1. Genetics

Recent advanced genetic and transcriptomic studies challenge a direct causative role of HCV in IPF. A 2024 bidirectional Mendelian randomization study demonstrated no causal relationship between chronic HCV infection and IPF at the genetic level, minimizing the impact of confounding factors [74].

#### 6.1.2. Direct Infection

Additionally, next-generation RNA sequencing of lung biopsies from IPF patients failed to detect significant HCV RNA expression, suggesting the virus is not directly residing in or chronically infecting the fibrotic lung tissue itself [75]. Despite the lack of direct genetic causation or active viral replication within the lung parenchyma, chronic HCV infection may trigger pulmonary fibrosis indirectly through other several pathways.

#### 6.1.3. Subclinical Alveolitis

The potential pathogenesis of IPF as an extrahepatic manifestation of HCV infection is thought to be driven by an occult, virus-induced pulmonary inflammatory reaction leading to subclinical alveolitis, though the specific cellular mechanisms. Kubo et al. propose that HCV triggers lymphocytes (T helper/inducer subset of T cells (CD4+) and HLA-DR+, a marker of early T-cell activation with no change in T suppressor/cytotoxic cells and B cells). Also, the study showed eosinophil-driven alveolitis, noting that eosinophils release highly cytotoxic products capable of causing tissue damage and interstitial fibrosis characteristic of IPF [76]. Another study by Yamagushi also supports the finding of Kubo showing elevation of eosinophils, lymphocytes of the same T-cell and surface marker groups in chronic HCV infection. Furthermore, administration of interferon-alpha (IFN-α) did not reduce the overall numbers of lymphocytes or eosinophils in the bronchoalveolar (BAL) fluid; it successfully normalized the lymphocyte surface markers, bringing the activated HLA-DR levels back down to normal ranges, implying HCV infection itself is the likely trigger for lymphocytic alveolitis, and that IFN-α treatment does not adversely induce these pulmonary abnormalities [77]. In contrast, Idilman et al. suggest these progressive fibrotic changes are instead mediated by a significant influx of polymorphonuclear neutrophils, which are recognized markers of inflammatory pulmonary disorders [78]. These divergent cellular profiles across studies may be influenced by methodological variances in BAL protocols, geographic differences, and variations in HCV infection phase. To confirm subclinical alveolitis, a study utilized 99mTc-diethylenetriamine pentaacetic acid (99mTc-DTPA) aerosol inhalation scintigraphy, a simple, sensitive, and noninvasive method for assessing pulmonary epithelial membrane permeability, an early marker of alveolar or interstitial injury. In asymptomatic patients positive for hepatitis C antibodies, this technique demonstrated a significantly increased 99mTc-DTPA clearance rate despite normal imaging findings, supporting the presence of subclinical alveolitis [79].

#### 6.1.4. Epithelial–Mesenchymal Transition (EMT)

IPF is a progressive and severe lung disorder characterized by excessive extracellular matrix deposition and fibrosis following alveolar injury, reflecting impaired epithelial repair mechanisms [80]. A central feature of this process is the epithelial–mesenchymal transition (EMT), during which alveolar epithelial cells lose polarity and cell–cell adhesion while acquiring mesenchymal morphology. This transition is marked by reduced expression of epithelial markers such as E-cadherin and zonula occludens-1 (ZO-1), alongside increased expression of mesenchymal markers including α-smooth muscle actin, vimentin, and N-cadherin [81]. EMT contributes to expansion of the myofibroblast population and promotes a profibrotic environment enriched with transforming growth factor-beta (TGF-β) and inflammatory cytokines. Although the pathogenesis of IPF is multifactorial, involving genetic and environmental factors, viral infections have also emerged as important contributors by disrupting epithelial signaling, sustaining inflammation, and promoting EMT. In particular, HCV has gained attention due to its potential not only in liver cirrhosis, but also its role in pulmonary fibrosis, as viral proteins can directly interact with key EMT-related pathways, including modulation of TGF-β/SMAD3 and NF-κB signaling, thereby influencing the expression of critical molecules including downregulation of E-cadherin and upregulation of fibroblast activation [82].

#### 6.1.5. Immune Complex Mediation

The impaired hepatic function in patients with HCV reduces the clearance of circulating antigens and antibodies, leading to the accumulation of immune complexes in the bloodstream and their deposition in various tissues, including the pulmonary vasculature [80]. These circulating immune complexes can directly promote fibroblast proliferation and neutrophil recruitment through macrophage activation, driving inflammation and subsequent fibrotic remodeling of lung tissue [82,83]. Additionally, these complexes may contribute to the development of mixed cryoglobulinemia and, in some cases, IPF as a secondary consequence of this [82]. A previous study using immunofluorescence microscopy did not detect immunoglobulin deposition in formalin-fixed lung tissue, whereas such deposition was identified in the kidneys. This finding suggests that circulating immunoglobulins may play only a limited role in the pathogenesis of IPF. However, use in formalin-fixed lung tissue might decrease the sensitivity of detection [71].

#### 6.1.6. Treatment (IFN-α)

Pharmacologic treatment of HCV may also act as a confounding factor in the development of IPF. IFN-α, previously used in HCV therapy, has been associated with a range of well-known pulmonary complications, including interstitial pneumonitis, sarcoidosis, and exacerbation of preexisting lung disorders. Although the exact incidence of IFN-α-induced interstitial pneumonia is uncertain, it has been estimated at approximately 0.3% in Japan and 0.01% in other regions [80]. The underlying mechanism is not fully understood but is thought to relate to its immunomodulatory effects [80,84]. Even though IFN-α can suppress regulatory T-cell activity and enhance cytotoxic T-cell responses, it might also lead to sustained immune activation and increased production of proinflammatory and profibrotic cytokines, which may predispose to pulmonary fibrosis [68,80,84]. However, an earlier study comparing patients before and after IFN therapy suggested that, despite its potential risks, IFN-α treatment for chronic HCV did not significantly worsen pulmonary parameters; in fact, it was better in normalizing T-cell surface markers [77].

The relationship between HCV and pulmonary fibrosis is complex and remains biologically plausible but unproven. While epidemiological studies show a strong association in certain geographic populations, recent genetic analyses argue against a direct causal link. Instead, the pathogenesis is likely multifactorial, driven by a combination of systemic immune activation, local subclinical alveolar inflammation, and questionable treatment-related outcomes.

## 7. Conclusions

Pulmonary complications of cirrhosis represent a complex consequence of altered liver–lung communication. HPS, PoPH, and HH arise through distinct mechanisms, yet all are influenced by portal hypertension, portosystemic shunting, impaired hepatic clearance, immune dysregulation, and changes in cardiopulmonary physiology. HPS is characterized primarily by IPVD and gas exchange impairment, primarily driven by increased vasoactive mediators like nitric oxide and altered angiogenesis. Conversely, PoPH is characterized by elevated pulmonary vascular pressure and resistance, resulting from hyperdynamic circulatory states and progressive pulmonary vascular remodeling, ultimately leading to right heart dysfunction. HH, a transudative pleural effusion, develops through movement of ascitic fluid across diaphragmatic defects into the pleural space and may cause substantial respiratory symptoms even in the absence of obvious ascites. Because these disorders often lack specific clinical manifestations apart from dyspnea, a high index of suspicion is required in patients with cirrhosis presenting with unexplained respiratory symptoms. Following exclusion of coexisting cardiac and primary pulmonary diseases, screening for these complications is particularly important in patients being evaluated for liver transplantation. Overall, early recognition, appropriate cardiopulmonary evaluation, and multidisciplinary management are essential, particularly because these complications affect treatment decisions, transplant prioritization, and long-term outcomes. Given that liver transplantation remains the only definitive treatment for many of these conditions, a thorough understanding of their pathophysiology is critical for timely diagnosis and optimal patient care.

Additionally, while chronic HCV infection frequently coincides with IPF in certain populations, current evidence suggests that this association is likely mediated through indirect and multifactorial mechanisms. Proposed pathways include subclinical alveolitis, systemic immune activation, and circulating immune complexes rather than simple direct viral infection of the lung parenchyma. The contribution of genetic susceptibility also remains uncertain and requires further investigation.

Moving forward, future research should focus on elucidating the precise molecular, immunologic, and microbiological drivers of pulmonary complications in liver disease. Identifying patients at risk of HPS and PoPH remains challenging because these conditions share several pathogenic pathways despite their distinct clinical and hemodynamic manifestations. Consequently, there is a pressing need for studies aimed at discovering reliable biomarkers and risk-stratification tools that can detect HPS, PoPH, or HH before the onset of advanced clinical disease and to help optimize the timing of liver transplantation and other therapeutic interventions. Increasing attention has been directed toward the gut–liver–lung axis as a potential contributor to the pathogenesis of these disorders. Although alterations in the gut microbiome have been shown to influence systemic immunity and have been implicated in pulmonary arterial hypertension, their specific role in the development of PoPH remains largely unexplored. Longitudinal investigations of gut–liver–lung signaling pathways are therefore warranted to better define their mechanistic role and therapeutic potential. Finally, regarding HCV-associated pulmonary fibrosis, well-designed prospective studies involving carefully characterized populations are needed to clarify the contribution of indirect immune-mediated mechanisms and to further elucidate the multifactorial pathogenesis underlying this relationship.

## Figures and Tables

**Table 1:** Characteristics of hepatopulmonary syndrome, portopulmonary hypertension, and hepatic hydrothorax.

	Hepatopulmonary Syndrome	Portopulmonary Hypertension	Hepatic Hydrothorax
Prevalence in chronic liver disease	13–56%	2–6%	5–16%
Pathogenesis	− Vasodilatation − Angiogenesis and intrapulmonary shunt − Vasculopathy	− Shear stress and vascular remodeling − Vasoconstriction	Transudative pleural effusion: − Movement of ascitic fluid into the pleural space through diaphragmatic defects − Hypoalbuminemia
Pathophysiology	− V/Q mismatch − Diffusion defect − Shunt	Pulmonary hypertension	Compression of lungs tissue
Clinical manifestation	Dyspnea, platypnea, orthodeoxia, spider nevi, cyanosis, digital clubbing	Dyspnea, peripheral edema, ascites, chest pain, syncope, right-sided heart failure	Dyspnea, non-productive cough, pleuritic chest pain
Diagnosis	Presence of liver disease or portal hypertension Impaired gas exchange (PaO_2_ ≤ 80 mmHg) with an elevated alveolar–arterial oxygen gradient ≥ 15 mmHg or ≥20 mmHg in patients > 64 years of age, in upright position Evidence of IPVD − Contrast-enhanced TTE − Radionucleotide lung perfusion scanning	Measured by TTE and RHC − mPAP > 20 mmHg − Pulmonary artery wedge pressure < 15 mmHg − PVR > 2	− Diagnostic thoracocentesis: Light’s criteria and serum-to-pleural fluid albumin gradient > 1.1 g/dL − Nuclear scans: tracer migrates into the chest cavity
Treatment	Liver transplantation	− PAH-specific medication − Liver transplantation if no contraindication	− Salt restriction − Diuresis − Therapeutic thoracocentesis − Pleurodesis − TIPS − Liver transplantation

IPVD, intrapulmonary vasodilatation; mPAP, mean pulmonary arterial pressure; PAH, pulmonary arterial hypertension; PaO_2,_ partial pressure of arterial oxygen; PVR, pulmonary vascular resistance; RHC, right heart catheterization; TIPS, transjugular intrahepatic portosystemic shunt; TTE, transthoracic echocardiography; V/Q, ventilation–perfusion.

## Data Availability

No new data were created or analyzed in this study. Data sharing is not applicable to this article.

## Abbreviations

The following abbreviations are used in this manuscript:

BAL	Bronchoalveolar
BMP	Bone morphogenetic protein
CT	Computed tomography
eNOS	Endothelial nitric oxide synthase
EMT	Epithelial–mesenchymal transition
ERA	Endothelin receptor antagonist
ET-1	Endothelin-1
FC	Functional class
HCV	Hepatitis C virus
HH	Hepatic hydrothorax
HO-1	Heme oxygenase-1
HPS	Hepatopulmonary syndrome
IL	Interleukin
IFN-α	Interferon-alpha
iNOS	Inducible nitric oxide synthase
IPF	Idiopathic pulmonary fibrosis
IPVD	Intrapulmonary vasodilatation
MASLD	Metabolic dysfunction-associated steatotic liver disease
MELD	Model for End-Stage Liver Disease
mPAP	Mean pulmonary artery pressure
MRI	Magnetic resonance imaging
NO	Nitric oxide
PAH	Pulmonary arterial hypertension
PaO_2_	Partial pressure of arterial oxygen
PAP	Pulmonary artery pressure
PDE5i	Phosphodiesterase-5 inhibitor
PDGF	Platelet-derived growth factor
PlGF	Placental growth factor
PoPH	Portopulmonary hypertension
PVR	Pulmonary vascular resistance
RNA	Ribonucleic acid
RVSP	Right ventricular systolic pressure
SBEM	Spontaneous bacterial empyema
TIPS	Transjugular intrahepatic portosystemic shunt
TNF-α	Tumor necrosis factor-alpha
TRV	Tricuspid regurgitation velocity
VEGF	Vascular endothelial growth factor
vWF	von Willebrand factor
V/Q	Ventilation and perfusion
ZO-1	Zonula occludens-1
6MWD	6 min walk distance
99mTc-DTPA	99mTc-diethylenetriamine pentaacetic acid (99mTc-DTPA)
99mTc-MAA	Technetium-99m macroaggregated albumin
